# Effect of *Rhizophagus irregularis* on Growth and Quality of *Cannabis sativa* Seedlings

**DOI:** 10.3390/plants10071333

**Published:** 2021-06-29

**Authors:** Ioanna Kakabouki, Antonios Mavroeidis, Alexandros Tataridas, Angeliki Kousta, Aspasia Efthimiadou, Stella Karydogianni, Nikolaos Katsenios, Ioannis Roussis, Panayiota Papastylianou

**Affiliations:** 1 Laboratory of Agronomy, Department of Crop Science, Agricultural University of Athens, 11855 Athens, Greece; antoniosmauroeidis@gmail.com (A.M.); a.tataridas@gmail.com (A.T.); aggelikh.kousta@gmail.com (A.K.); stella.karidogianni@hotmail.com (S.K.); iroussis01@gmail.com (I.R.); ppapastyl@aua.gr (P.P.); 2 Department of Soil Science of Athens, Institute of Soil and Water Resources, Hellenic Agricultural Organization—Demeter, Sofokli Venizelou 1, Lycovrissi, 14123 Attica, Greece; sissyefthimiadou@gmail.com (A.E.); nkatsenios@gmail.com (N.K.)

**Keywords:** *Rhizophagus irregularis*, *Glomus intraradices*, *Cannabis sativa* L., colonization, plant growth, seedling quality, Dickson’s quality index

## Abstract

*Rhizophagus irregularis* is an arbuscular mycorrhiza fungus that can enhance plant nutrition and reduce transplant shock on seedlings. The present study aims to evaluate the effects of this fungus on the quality of cannabis (*Cannabis sativa* L.) seedlings. A greenhouse float system experiment was conducted in a completely randomized design with three treatments. The treatments included the application of 40, 80 and 120 fungus spores per L of nutrient solution (AMF1, AMF2 and AMF3, respectively). The evaluation was performed based on the agronomic characteristics of the seedlings (root and stem length and weight, stem diameter), N and P content, survival rate, and the Dickson’s quality index (DQI). Results indicated that root length and stem dry weight were significantly increased (by 34.14% and 21.4%, respectively) in the AMF3 treatment. The biomass of the seedlings’ roots, the fresh weight and the N content were not affected by the AMF. On the contrary, survival rate, P content and DQI were significantly increased in AMF3 (by 5%, 24.3% and 12.4% respectively). Overall, our findings suggest that the application of high doses of *Rhizophagus irregularis* (AMF3) on float system-produced cannabis seedlings results in a considerable increment of their quality.

## 1. Introduction

Cannabis (*Cannabis sativa* L.) is one of the earliest domesticated crops that has been cultivated for centuries due to its adaptability to various environments [1,2]. Its cultivation is linked with numerous products in textile fiber, food, seed oil, and medicine sectors [3,4]. Cannabis produces textiles of high quality and great strength [5], and seeds of high nutraceutical value, while it composes hundreds of secondary metabolites with medicinal components [4,6]. Despite the versatility of cannabis, legal restrictions led to the prohibition of the crop due to the psychoactive secondary metabolite Δ^9^-tetrahydrocannabinol (THC) [7]. However, during recent decades significant interest has been raised due to the reauthorization of the crop [8,9,10,11]. Nowadays, cannabis is cultivated in more than 40 countries worldwide, as well as in several European Union (EU) countries [12].

Even though the ban of the crop caused a lack of science-based information considering agronomic practices, a wide range of agricultural systems have been developed in cannabis production [10]. Amongst them, hydroponic soilless cannabis cultivation has been proved to be equally as successful as the conventional practice on the soil [13], or occasionally might be even better [14]. Hydroponic systems, such as the float system, are frequently used for the production of plant seedlings including vegetables, tobacco, and cannabis [15,16,17]. In a standard float system, the cultivation is usually performed on Styrofoam sheets that float on top of basins filled with nutrient solutions [18]. Nutrient solutions can be either organic- or inorganic-based and impact the pant growth [19]. It is worth mentioning that plants produced on float systems usually demonstrate a significantly increased root system [20]. Since the length, diameter, and density of the roots are directly correlated with the plants’ water and nutrient uptake potential [21,22], float system seedlings present high-quality characteristics [23]. Dickson’s Quality Index (DQI) through root biomass and other agronomic characteristics determination can be utilized for the evaluation of the seedling quality [24].

Even though cannabis is a low-input crop, it shows high demands for essential nutrients such as nitrogen [8]. In cannabis cultivars for fiber production, it is reported that the supply of high nitrogen levels increased leaf weight, while the THC content was reduced [25]. In terms of the pharmaceutical components of cannabis, phosphorus is crucial for the production of secondary metabolites such as cannabinoids [26,27]. Nutrient deficiency could lead to irregular growth symptoms and subsequently to yield losses [28].

The addition of Plant Growth Promoting Microorganisms (PGPMs) in nutrient solutions, in float systems, could be beneficial for cannabis seedlings. Recently, studies indicated the positive impact of PGPMs on seedlings of various plant species [29,30,31,32]. PGPMs improve plant nutrition and affect the release of biologically active substances such as phytohormones, vitamins, and enzymes [33,34]. Using important processes such as the dissolution of soluble minerals and nitrogen stabilization, PGPMs facilitate the nutrients uptake by plants [33]. In the case of cannabis, PGPMs such as *Trichoderma harzianum* has been found to boost the growth of the plants and affect their agronomic characteristics [35].

Arbuscular Mycorrhizae Fungi (AMF) are an example of PGPMs that can form symbiotic relationships with plants [36]. The AMF, through these relationships, can reportedly improve crop performance [37]. Various fungi species of the Glomaceae family are included in the category of AMF [38]. Particularly, research has been performed regarding the effects of *Rhizophagus irregularis* (formerly *Glomus intraradices*) on satureja, soybean, and basil cultivations [39,40,41]. In the context of these studies, the presence of these fungi on plant roots equilibrated the adverse effect of abiotic stress and improved plant nutrition.

Although there is available literature about the combined use of AMF and *Trichoderma harzianum* on cannabis, there is a lack of data on *Rhizophagus irregularis* effects in cannabis seedlings. The present study aims to examine for the first time to our knowledge, the effect of *Rhizophagus irregularis* on the quality of cannabis seedlings produced on a float system, by utilizing Dickson’s Quality Index.

## 2. Results

### 2.1. Mycorrhizal Colonization

**Figure 1:**
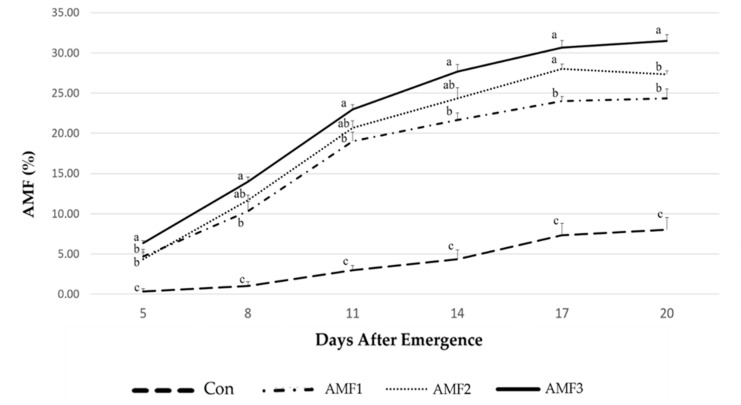
Percentage of AMF colonization derived from the three different treatments of AMF, 20 days after the emergence of the seedlings. F-test ratios are from ANOVA. Different letters (a, b, and c) over each curve indicate significant differences according to Tukey’s test (*p* < 0.05).

Root colonization analysis demonstrated that control treatment reported the lowest mycorrhizal infestation. As depicted in Figure 1, the AMF colonization on the non-treated cannabis plants was maintained below 10% during the first fourteen days after seedling emergence. Significant differences were noted among treatments, whereas the addition of the AMF seems to augment root colonization. Among the three different treatments of AMF, the maximum dose (AMF3) favored the colonization to a greater extent, succeeding 30%. On the contrary, AMF1 and AMF2 did not significantly affect the colonization.

### 2.2. Plant Growth

The presence of the AMF affected both the above and the underground tissues of cannabis plants (Table 1). Notable differences were observed between treated and untreated plants (*p* < 0.05), regarding the total root length, after the application of AMF. Specifically, the total root length of the AMF-treated plants was increased by 34.14% compared to the control. As for the root dry mass, no statistically significant differences were noted.

Regarding the aboveground cannabis growth, the addition of AMF does not benefit the plant height, shoot fresh weight, or the shoot diameter. On the contrary, the dry stem weight was increased with the augmentation of AMF. Consequently, the higher values of dry stem weight were recorded after AMF3 application, while significant differences were noticed between the non-treated and the treated cannabis plants (*p* < 0.01).

The ratio of dry stem mass to root dry mass (DSW/RDM) and the stem diameter did not demonstrate statistically significant differences. Nevertheless, the increased rates of *Rhizophagus irregularis* favored DSW/RDM. Moreover, the total dry weight of aboveground and underground cannabis tissues is enhanced by increased AMF doses, recording significant differences (*p* < 0.001). The highest values were in AMF3 plants (3.20 g), while the lowest values were related to Control plants (3.73). Therefore, the results of our study are in line with the assumption that AMF colonization contributes to plant growth enhancement.

The P uptake was positively correlated to AMF (*p* < 0.05). Specifically, significant differences were recorded between the Control and AMF3 plants (Table 2), where the highest values were observed (0.46 mg/kg). Regarding the N content of the plants, AMF did not lead to a significant increase. However, the N content was greater in AMF3 plants (2.58%).

The AMF3 positively affected the survival rate of the seedlings as it was increased by 5%, compared to the control (Table 2). Similarly, significant differences were noted in the DQI, where the highest values were recorded in the AMF3 treatment (0.1218).

## 3. Discussion

Colonization with AMF is known to play a key role in the promotion of plant growth and the quality of plant seedlings [42]. The ability of AMF to stimulate plant growth can be observed through various parameters and in different plant tissues, such as biomass accumulation [43,44]. These effects of AMF colonization on plants were well depicted in our experiment after the treatments with *Rhizophagus irregularis* on cannabis.

*Rhizophagus irregularis* enhanced the root growth of cannabis by increasing the total root length and the dry biomass. AMF3 increased total root length almost by 25%, compared to the control. A recent study confirms our results, reporting a positive impact of *Rhizophagus irregularis* on the root growth of basil, and the root biomass of satureja [41]. Moreover, compared to untreated plants, mycorrhizal colonization significantly increased both the dry stem weight and the total dry weight of the cannabis. Similar results have been noted on maize plants treated with *Rhizophagus irregularis* [45].

These results indicate that there is a strong positive correlation between AMF root colonization and plant growth parameters such as root development, and total dry biomass of cannabis seedlings (Table 3). That fact can become more evident, considering the rate of AMF root colonization. Specifically, in cases of higher rates of *Rhizophagus irregularis*, the infestation rate was even higher and led to a more intense symbiosis between AMF and the host plant. This mechanism benefits the strengthening of the root system and consequently advanced plant growth [46].

Interestingly, the mutualistic symbiosis in this study seems to affect the uptake of nutrients from seedlings. In the case of phosphorus (P), *Rhizophagus irregularis* ensured its availability in the rhizosphere and facilitated its assimilation by the seedlings. The highest *p* values were recorded in the highest dose of AMF (AMF3). Previous studies verify the beneficial effect of *Glomus* species on P uptake [42]. Even though phosphorus is not readily available to plants due to its low solubility, processes such as the mineralization by extracellular phosphatase and the solubilization by mycorrhizal fungi, make P available to seedlings [47,48]. Thus, P as a vital nutrient for plant metabolism and growth can be a component in essential physiological mechanisms [49]. P adequacy becomes even more imperative in plant species such as cannabis, where the reproductive organs and the inflorescence initiation might be negatively affected by P insufficiency [49]. As for the N accumulation, no significant increase was noticed after treating with AMF. This result might be attributed to the absorption of N via the extraradical hyphae from the soil. As a result, the nutrients were assembled in the rhizosphere instead of seedling leaves and stem. Chen et al. also observed similar results [50].

The application of *Rhizophagus irregularis* has also been found to be able to alter the photosynthetic rates of seedlings of various plant species [51,52,53]. Increased photosynthesis could be amongst the factors responsible for the observed increased biomass of the cannabis seedlings. Increased AMF-induced P availability could indirectly impact photosynthesis via affecting ATP/ADP ratios or by affecting RuBP carboxylase activity [54]. This hypothesis agrees with the increased P content of the seedlings, which we also observed. Moreover, according to Nemec and Vu [55], *Rhizophagus irregularis* can potentially increase PEPCase activity, an enzyme associated with the synthesis of malate. Malate is the end product of the carbon reduction cycle in C_3_ plants, such as cannabis, thus further validating that *Rhizophagus irregularis* affect photosynthesis.

It should be noted though, that better nutrition and increased photosynthetic rates might not be the sole reasons responsible for the overall improved survival rate and quality of the seedlings. Besides their effects on the nutrient uptake of the plants, Al-Arjani et al. reported that AMF enhance the production of phytohormones [56]. Similarly, according to the findings of Jaitieng et al. [57], the combined application of AMF (*Glomus intraradices* and *Glomus* sp.) and rock phosphate fertilizer on robusta coffee plants (*Coffea canephora*) caused a significantly augmented accumulation of salicylic acid in the leaves of the plants. Apart from its role in the stress-alleviating mechanisms, salicylic acid has been reported to influence seed germination and seedling establishment [58].

Furthermore, studies have also indicated that *Rhizophagus irregularis* can reduce the transplant shock on seedlings [59,60]. Krishna et al. [61] studied the effects of AMF (including fungi of the *Glomus* species) on the micro propagated grapevine (*Vitis vinifera* L.) and concluded that AMF reduce transplant shock. They also proposed that the accumulation of proline was responsible for the stress resistance, as they observed higher levels of this amino acid in AMF-treated plants. This hypothesis is supported by several studies regarding the importance of proline in osmotic adjustment and protection of proteins under stress conditions [62]. Regardless of the proline levels, transplant-shock endurance is widely attributed to well-developed roots, able to support the plant, withstand environmental changes and absorb adequate nutrients and water [63].

Although in the present study the accumulation of compounds such as salicylic acid and proline were not measured, AMF indeed enhanced root development. The potential involvement of AMF induced phytohormones, proteins, and other biological molecules in cannabis seedling quality should not be dismissed. Further research should be conducted regarding the interaction of *Rhizophagus irregularis*, its metabolites, and the cannabis biochemical pathways that they potentially trigger, that result in vigorous seedlings.

Regarding the DQI, the positive correlation between this index and AMF that was noted in our study (Table 3) is in line with most of the literature. DQI has been utilized in the past by researchers in their effort to evaluate the effects of AMFs on the seedling quality of various species [64,65,66]. Notably, DQI’s correlation with the agronomic characteristics of cannabis seedlings was not significant (Table 3), even though it is an indicator of robustness and plant biomass [67]. The total N content of the seedlings, though, seems to affect DQI (Table 3), implying that perhaps nutrition influences the values of this index. Regardless of this interaction, the results of this present study reveal that DQI can be utilized to objectively evaluate the quality of cannabis seedlings.

## 4. Materials and Methods

A greenhouse experiment was conducted at the Agricultural University of Athens (37°58′55.83″ N, 23°42′16.69″ E) during April 2019 to evaluate the effects of the mycorrhizal fungus *Rhizophagus irregularis* on the quality of cannabis seedlings. The experiment was set up in a completely randomized design with 4 treatments (Table 4) and 3 replications.

Initially, polystyrene trays with 150 cells (each cell’s volume was 12 cm^3^) were filled with organic peat. On each individual cell, 1 seed of *Cannabis sativa* (variety USO 31) was sown. The trays were then placed on 50 L basins filled with water, where 2 trays were placed on each basin. In all the basins, 1 L of Fish–Fert organic (N-P-K: 2-4-0.5) water-soluble fertilizer (Humofert Co., Athens, Greece) was added. Besides the untreated control, different doses of the commercial product MycoPlant^®^ Polvo Grow by TRATAMIENTOS BIO-ECOLOGICOS (containing water-dispersible granules of *Rhizophagus irregularis*) were also added in each basin. The number of fungus spores added in each basin can be seen in Table 4. The seedlings emerged 7 days after sowing (DAS) and were transplanted in 12 L pots, filled with a mixture of soil and compost (1:1 *v/v*), 35 days after their emergence (DAE).

The parameters that were considered to evaluate the seedlings were their agronomic characteristics, their nitrogen (N) and phosphorus (P) content, and their survival rate. Measurements regarding the first two parameters required destructive sampling. Therefore, 20 DAE, 5 plants per treatment were collected as samples for the assessment of their agronomic characteristics. Measurements included the recording of the fresh and dry weight of their roots and stems, the diameter of stems, the total root length, and the height of the plants. For the valuation of the dry weight, the samples were first placed in an oven at 70 °C for 3 days.

The length and diameter of the roots were measured by a high-resolution Delta-T Scan, version 2.04 (Delta Devices Ltd., Burwell, Cambridge, UK). These measurements were then further utilized to calculate the Dickson’s quality index (DQI), according to the Equation (1):

(1) $$\mathrm{DQI}=\frac{Seedling\;total\;dry\;weight\;\left(g\right)}{\frac{Shoot\;lenght\;\left(mm\right)}{Stem\;diameter\;\left(mm\right)}+\frac{Shoot\;dry\;weight\;\left(g\right)}{Root\;dry\;weight\;\left(g\right)}}$$

A nutrient analysis was performed to determine the N and P content of the seedlings. The total N content was determined by applying the Kjeldahl method on the dried samples. For the determination of the P content (mg kg^−1^), 5 additional plants per treatment were collected, dissolved in an HNO_3_-H_2_O_2_ solution, and heated under pressure in a CEM MDS 2000 microwave (CEM Ltd., Buckingham, UK). The extract was then analyzed by an iCAP 6500 DUO ICP-emission spectrometer (Thermo Fisher Scientific, Waltham, MA, USA). The survival rate was visually estimated 40 DAE. Finally, the colonization of the root system by the Arbuscular Mycorrhizal Fungi (AMF) also measured 5, 8, 11, 14, 17, and 20 DAE according to the Gridline section method at 40 × magnification [68].

For the analysis of variance, the STATISTICA v10 (StatSoft, Inc., Tulsa, OK, USA, 2011) logistic package was used. Significant differences amongst treatments were determined using Fisher’s least significant difference test (LSD) at the 5% level of probability (*p* < 0.05).

## 5. Conclusions

In the present study, the supply of *Rhizophagus irregularis* in nutrient solutions of float system seems to enhance the growth and quality of cannabis (*Cannabis sativa* L.) seedlings. The presence of *Rhizophagus irregularis* as a PGPM affected the growth parameters of both aboveground and underground cannabis tissues. In particular, the total root length, and the total biomass of cannabis were significantly increased. Furthermore, this mutualistic symbiosis positively influenced nutrient uptake by the seedlings. Significant differences were recorded in the accumulation of phosphorus by seedlings in the AMF3 treatment, while the N content was not significantly increased. Moreover, *Rhizophagus irregularis* enhanced the stress resistance of seedlings recording a notable increment of survival rate. Although AMF3 led to seedlings of better quality, further research should be conducted regarding the mechanisms and the biochemical pathways behind the beneficial interaction between *Rhizophagus irregularis* and cannabis seedlings.

## Figures and Tables

**Table 1:** Plant characteristics of aboveground and underground growth as affected by different treatments of AMF.

	Total Root Length (cm)	Root Dry Mass (g)	Plant Height (cm)	Fresh Weight (g)	Dry Stem Weight (g)	DSW/RDM	Stem Diameter (cm)	Total DW (g)
Control	207 ^a^	1.19 ^ns^	11.54 ^ns^	4.97 ^ns^	2.01 ^a^	1.70 ^ns^	0.40 ^a^	3.20 ^a^
AMF1	245 ^ab^	1.23 ^ns^	11.23 ^ns^	4.74 ^ns^	2.20 ^b^	1.80 ^ns^	0.37 ^b^	3.43 ^bc^
AMF2	258.33 ^bc^	1.26 ^ns^	11.45 ^ns^	4.80 ^ns^	2.30 ^bc^	1.83 ^ns^	0.38 ^b^	3.56 ^b^
AMF3	277.67 ^c^	1.29 ^ns^	11.69 ^ns^	4.99 ^ns^	2.44 ^c^	1.89 ^ns^	0.40 ^a^	3.73 ^c^
F	6.13 *	1.08 ^ns^	0.39 ^ns^	1.71 ^ns^	19.70 ***	1.37 ^ns^	3.96 *	13.48 **

F-test ratios are from ANOVA. Different letters (a, b and c) within a column indicate significant differences according to Tukey’s test. Significance levels: * *p* < 0.05; ** *p* < 0.01; *** *p* < 0.001; ns, not significant (*p* > 0.05).

**Table 2:** The survival rate, seedling index, and nitrogen and phosphorus accumulation as affected by different treatments of AMF.

	Survival Rate	N Content %	P Content (mg/kg)	Seedling Index (DQI)
Control	90.83 ^a^	2.26 ns	0.37 a	0.1084 a
AMF1	92.93 a	2.45 ns	0.41 a	0.1088 ab
AMF2	93.60 a	2.46 ns	0.43 a	0.1138 ^bc^
AMF3	95.53 b	2.58 ns	0.46 b	0.1218 c
F	4.20 *	0.73 ns	4.15 *	7.18 *

F-test ratios are from ANOVA. Different letters (a, b and c) within a column indicate significant differences according to Tukey’s test. Significance levels: * *p* < 0.05; ns, not significant (*p* > 0.05).

**Table 3:** Correlation matrix between agronomic characteristics, nutrient content, AMF percentage and Dickson’s quality index.

	Total Root Length (cm)	Root Dry Mass (g)	Plant Heigh (cm)	Fresh Weight (g)	Dry Stem Weight (g)	DSW/RDM	Stem Diameter (cm)	Total DW	Survival Rate	N Content %	P Content mg/kg	Seedling Index (DQI)
**AMF%20 DAE**	0.8233 *	0.4935 ^ns^	−0.0227 ^ns^	0.1564 ^ns^	0.9019 ***	0.5607 ^ns^	−0.1119 ^ns^	0.8689 ***	0.7319 ^ns^	0.6861 ^ns^	0.7660 *	0.7584 *
**Seedling Index**	0.5569 ^ns^	0.4462 ^ns^	−0.2340 ^ns^	0.4793 ^ns^	0.6476 ^ns^	0.3137 ^ns^	0.4291 ^ns^	0.6560 ^ns^	0.4873 ^ns^	0.7977 *	0.4205 ^ns^	-

Significance levels: * *p* < 0.05; *** *p*< 0.001; ns, not significant (*p* > 0.05).

**Table 4:** Treatments and total *Rhizophagus irregularis* applied rates.

Treatment	MycoPlant^®^ Polvo Grow Dose (g L^−1^)	Total Applied Fungus Spores
Control	-	-
AMF1	0.1	2000
AMF2	0.2	4000
AMF3	0.3	6000

## Data Availability

The data presented in this study are available on request from the corresponding author.

## Abbreviations

AMF, arbuscular mycorrhizal fungi; N, nitrogen; P, phosphorus; DQI, Dickson’s quality index; THC, Δ9-tetrahydrocannabinol; EU, European Union; PGPMs, plant growth promoting microorganisms; DAS, days after sown; DAE, days after emergence.
